# Structural network disruption of corticothalamic pathways in cerebral small vessel disease

**DOI:** 10.1007/s11682-024-00889-4

**Published:** 2024-05-08

**Authors:** Xuejia Jia, Yingying Li, Xiuqin Jia, Qi Yang

**Affiliations:** 1 Department of Radiology, Beijing Chaoyang Hospital, Capital Medical University, Beijing, 100020 China; 2 Key Lab of Medical Engineering for Cardiovascular Disease, Ministry of Education, Beijing, 100020 China; 3 Laboratory for Clinical Medicine, Capital Medical University, Beijing, 100020 China

**Keywords:** Cerebral small vessel disease, Structural network, Cognition

## Abstract

Generalized fractional anisotropy (GFA) can eliminate the crossing fiber effect, which may be more reflective of brain tissue changes in patients with cerebral small vessel disease (CSVD). This study aimed to explore the alterations of structural networks based on GFA and its relationship with cognitive performance in CSVD patients. We recruited 50 CSVD patients which were divided into two groups: cognitive impairment (CSVD-CI) and normal cognition (CSVD-NC), and 22 healthy controls (HCs). All participants underwent the Montreal Cognitive Assessment (MoCA) and MRI examinations. The structural topological properties were compared among the three groups. The correlation between these structural alterations and MoCA was analyzed. Compared with HCs, significantly decreased nodal efficiency and connectivity were detected in the corticothalamic pathways in both patient groups, of which some were significantly decreased in CSVD-CIs compared with CSVD-NCs. Moreover, both patient groups exhibited global network disruption including decreased global efficiency and increased characteristic path length compared with HCs. Furthermore, the nodal efficiency in the right pallidum positively correlated with MoCA in CSVD-NCs controlling for nuisance variables (*r* = 0.471, *p* = 0.031). The alterations in corticothalamic pathways indicated that the brain structural network underwent extensive disruption, providing evidence for the consideration of CSVD as a global brain disease.

## Introduction

Cerebral small vessel disease (CSVD) is the major cause of vascular cognitive decline and dementia (Jellinger, 2007; Pantoni, 2010). It refers to a series of clinical, imaging, and pathological syndromes caused by various etiologies affecting small blood vessels in the brain (Ter Telgte et al., 2018). Currently, the diagnosis of CSVD relies mainly on conventional imaging. However, the subtle changes in brain structure may occur early before the initial onset of conventional neuroimaging features.

Ed Bullmore et al. highlighted the growing relevance of graph theory analysis approaches in understanding the physics of brain connectome (Bullmore & Sporns, 2009). In particular, structural connectivity exhibits greater sensitivity than functional connectivity when assessing nodal and global network parameters (Zhu et al., 2022), and it is also considered as a predictor of functional connectivity (Bullmore & Sporns, 2009).

Advances in diffusion tensor imaging (DTI) have enabled the examination of microstructural changes in white matter, preceding the occurrence of visible lesions on conventional magnetic resonance imaging (MRI) in patients with CSVD. Specifically, fractional anisotropy (FA) is the most widely used diffusion parameter. However, FA is limited to representing a single fiber direction (Alexander et al., 2001), thus leading to an underestimation of the influence of crossing fibers (Winston, 2012). The emerging diffusion parameter generalized fractional anisotropy (GFA), derived from the q-ball imaging linear reconstruction scheme, addresses the problem of fiber crossing in deep white matter pathways and subcortical margins in vivo (Tuch, 2004). It is considered to be robust and stable compared with FA (Ji et al., 2019; Ueda et al., 2016).

We aimed to explore the structural topological alterations in patients with CSVD using GFA and to examine the relationship between these alterations and cognitive performance in CSVD patients. In the present study, it was hypothesized that the corticothalamic pathways might exhibit structural alterations in patients with CSVD.

## Materials and methods

### Participants

We enrolled 50 asymptomatic CSVD patients that met the following criteria: (a) age ≥ 50 years, and (b) one or more CSVD neuroimaging biomarkers on MRI with CSVD score ≥ 1. Neuroimaging biomarkers included white matter hyperintensity (WMH) of presumed vascular, recent small subcortical infarcts, lacune, perivascular space (PVS), cerebral microbleed (CMB) and brain atrophy (Duering et al., 2023; Wardlaw et al., 2013). The CSVD score was an assessment of the total MRI burden for CSVD neuroimaging biomarkers, allocating 1 point each for the presence of: (a) deep WMH (Fazekas score ≥ 2) or periventricular WMH (Fazekas score = 3), (b) one or more lacunes, (c) one or more CMBs, and (d) moderate to severe PVS in the basal ganglia region (grade 2–4) (Smith et al., 2012; Staals et al., 2014). In addition, 22 age- and sex-matched healthy controls (HCs) were included from the community.

The exclusion criteria were as follows: (a) history of genetic CSVD and cerebral amyloid angiopathy, (b) participants with transient ischemic attack within 3 months, (c) history of intracranial atherosclerotic disease, (d) history of psychiatric disorders, (e) other neurological diseases, such as multiple sclerosis and Parkinson disease, (f) intracranial hemorrhage or occupying lesions, (g) visual or auditory impairment, and (h) claustrophobia or other contraindications to MRI.

All participants underwent MRI examinations and the Montreal Cognitive Assessment (MoCA). The MoCA score was corrected for education level, increasing by 1 point when the education level of the participant was ≤ 12 years, with a maximum score of 30. The patients were divided into two groups: 29 CSVD patients with cognitive impairment (CSVD-CI) and 21 CSVD patients with normal cognition (CSVD-NC), using a cutoff MoCA score of 26 (Nasreddine et al., 2005).

This study was approved by the Ethics Committee Boards of Beijing Chaoyang Hospital, Capital Medical University and written informed consent was obtained from each participant.

### Image acquisition

The participants underwent brain MRI on a 3.0 T scanner (MAGNETOM Skyra, Siemens Healthcare) with a 20-channel head coil. The T1-weighted magnetization-prepared rapid acquisition gradient echo (MP-RAGE) imaging parameters were: repetition time (TR) = 2300 ms, echo time (TE) = 2.32 ms, inversion time (TI) = 450 ms, slice thickness = 1 mm, gap = 50%, flip angle = 8°, field of view (FOV) = 256 × 256 mm^2^, number of slices = 192. The T2 imaging parameters were: TR = 3900 ms, TE = 99 ms, slice thickness = 5 mm, number of slices = 20. T2-weighted fluid attenuated inversion recovery (FLAIR) imaging parameters were: TR = 8000 ms, TE = 81 ms, slice thickness = 5 mm, number of slices = 20. DTI was acquired in the anterior-to-posterior phase-encoding direction, and b-value for non-zero gradient volumes was 1000 s/ mm^2^ along 64 gradient directions: acquisition matrix = 74 × 74, axial slices = 76, TR = 5200 ms, TE = 74 ms. Susceptibility weighted imaging (SWI) parameters were: TR = 28 ms, TE = 20 ms, slice thickness = 2 mm, FOV = 220 × 220 mm^2^. 

### Data preprocessing

Data preprocessing was performed using DSI Studio (http://dsi-studio.labsolver.org). First, the DTI data were corrected for motion and eddy current distortion. Then the images were reconstructed in the Montreal Neurological Institute (MNI) space using q-space diffeomorphic reconstruction, which were rotated and scaled to the space of T1MPRAGE with b-table also rotated accordingly. A deterministic fiber tracking algorithm was used with augmented tracking strategies to improve reproducibility (Yeh, 2020). A seeding region was placed at whole brain, where a total of 1,000,000 seeds were placed. The anisotropy threshold was randomly selected. The angular threshold was randomly selected from 15 degrees to 90 degrees and the step size was randomly selected from 0.5 voxel to 1.5 voxels. Tracks with length shorter than 10 or longer than 200 mm were discarded.

### Network node and edge definition

Automated anatomical labeling (AAL) atlas was used as the brain parcellation (Rolls et al., 2020), and the connectivity matrix was calculated using GFA and FA of the connecting tracks. For the network node definition, the whole brain was divided into 166 cortical or subcortical regions by the AAL3 atlas with each region as a node. We defined the average GFA and FA value of the white matter fibers between two nodes as the weight of the edge (Zhou et al., 2022).

### Network analysis

All network parameters, including nodal and global topological network feature parameters, were obtained using the graph theoretical network analysis (GRETNA) toolbox (https://www.nitrc.org/projects/gretna). In this study, weighted networks were chosen to identify subtle network organization (Martensson et al., 2018). Brain networks are often compared with random networks to test whether they are configured with a significant non-random topology. Random networks are generated by the Markov wiring algorithm (Maslov & Sneppen, 2002), which preserves the same number of nodes and edges and the same degree distribution as the real brain network. The sparse thresholds used in this study ranged from 0.1 to 0.3 with an interval of 0.01.

At the nodal level, we calculated nodal efficiency, the inverse of the shortest path length of a subnetwork in which the node participated. This quantified the importance of nodes for information communication within the network (Fox & King, 2018). In global level, we calculated the area under the curve (AUC) values of global network parameters and their values at various thresholds: (1) global efficiency (Eglob), measured the efficiency of parallel message transmission in the whole-brain network; (2) the characteristic path length (Lp), calculating the average shortest path length between all pairs of nodes in the network; (3) gamma (γ), normalized clustering coefficient; (4) lambda (λ), normalized Lp; (5) sigma (σ), small-worldness, γ/λ (Reijmer et al., 2013; Rubinov & Sporns, 2010; Wang et al., 2011).

### Statistical analysis

Demographics and clinical characteristics were analyzed using SPSS software (version 26.0, IBM). We assessed whether demographic data and global network parameters were statistically disparate among three groups. One-way analysis of variance (ANOVA) was chosen if continuous data followed a normal distribution and homogeneous variance, and Bonferroni post hoc tests were performed. In the case of non-normal or heterogeneous variance, Kruskal-Wallis tests were used. For categorical variables, chi-square tests were performed. We used a statistical significance level of *p* < 0.05.

One-way ANOVA was performed for nodal efficiency among the three groups to obtain the significantly different brain regions, which were defined as regions of interest (ROIs). We performed the false discovery rate (FDR) correction on the ROIs, using the built-in function in MATLAB: mafdr (p_vector,‘BHFDR’, true). Further least significant difference post-hoc tests were used for pairwise comparisons. In the CSVD-CI and CSVD-NC group, partial correlation and regression analyses were performed to test the relationship between nodal efficiency and MoCA for brain regions, considering age, sex, and education as covariates. The above analyses were performed in SPSS 26.0. The connectivity difference was calculated using the two-sample t-test followed by network-based statistics (NBS) correction with 5,000 permutations for each participant using the GRETNA toolbox. The significance level was set at *p* < 0.05 corrected.

## Results

### Demographic and clinical characteristics of participants

The demographic characteristics and clinical parameters of each group are summarized in Table 1. The CSVD-CI group had significantly decreased MoCA than the CSVD-NC and HC groups (*p* < 0.001). No significant differences in age, sex, and neuroimaging features were observed among the three groups.

**Table 1:** Demographic and clinical characteristics of all participants

Characteristics	CSVD-CI(*n* = 29)	CSVD-NC(*n* = 21)	HC(*n* = 22)	*p*
**Demographics**				
Age, year	59.69 ± 4.42	58.43 ± 5.24	58.14 ± 5.29	0.49
Male sex, no. (%)	18(62)	15(71)	9(41)	0.11
Education, year	9.00 ± 2.98	10.95 ± 3.28	13.09 ± 2.71	**<0.001** ^#*+^
MoCA	21(5)	28(3)	27(1)	**<0.001** ^#*^
**Vascular risk factors**				
Hypertension, no. (%)	21(72)	13(62)	8(36)	**0.03**
Diabetes, no. (%)	5(17)	6(29)	7(32)	0.45
Hypercholesterolemia, no. (%)	17(59)	10(48)	6(27)	0.08
Smoking, ever, no. (%)	18(62)	12(57)	1(5)	**<0.001**
Alcohol, ever, no. (%)	12(41)	7(33)	1(5)	**0.01**
Body mass index	31.41 ± 10.49	30.27 ± 8.51	27.15 ± 7.68	0.25
**Neuroimaging features**				
WMH Fazekas score	3(2)	3(1)	-	0.15
Lacunes, no. (%)	4(13)	2(10)	-	0.65
PVS	1(2)	1(1)	-	0.22
Microbleeds, no. (%)	10(34)	7(33)	-	0.93

***Note***: Values represented as mean ± SD, median (IQR), or number of participants (%)

^**#**^ indicates significant differences between CSVD-CI group and HC group

^*^ indicates significant differences between CSVD-NC group and HC group

^+^ indicates significant differences between CSVD-CI group and CSVD-NC group. CSVD-CI, CSVD patients with cognitive impairment; CSVD-NC, CSVD patients with normal cognition; HC, healthy control; MoCA, the Montreal Cognitive Assessment; PVS, perivascular space; WMH, white matter hyperintensity

### Node-based analysis

**Fig. 1:**
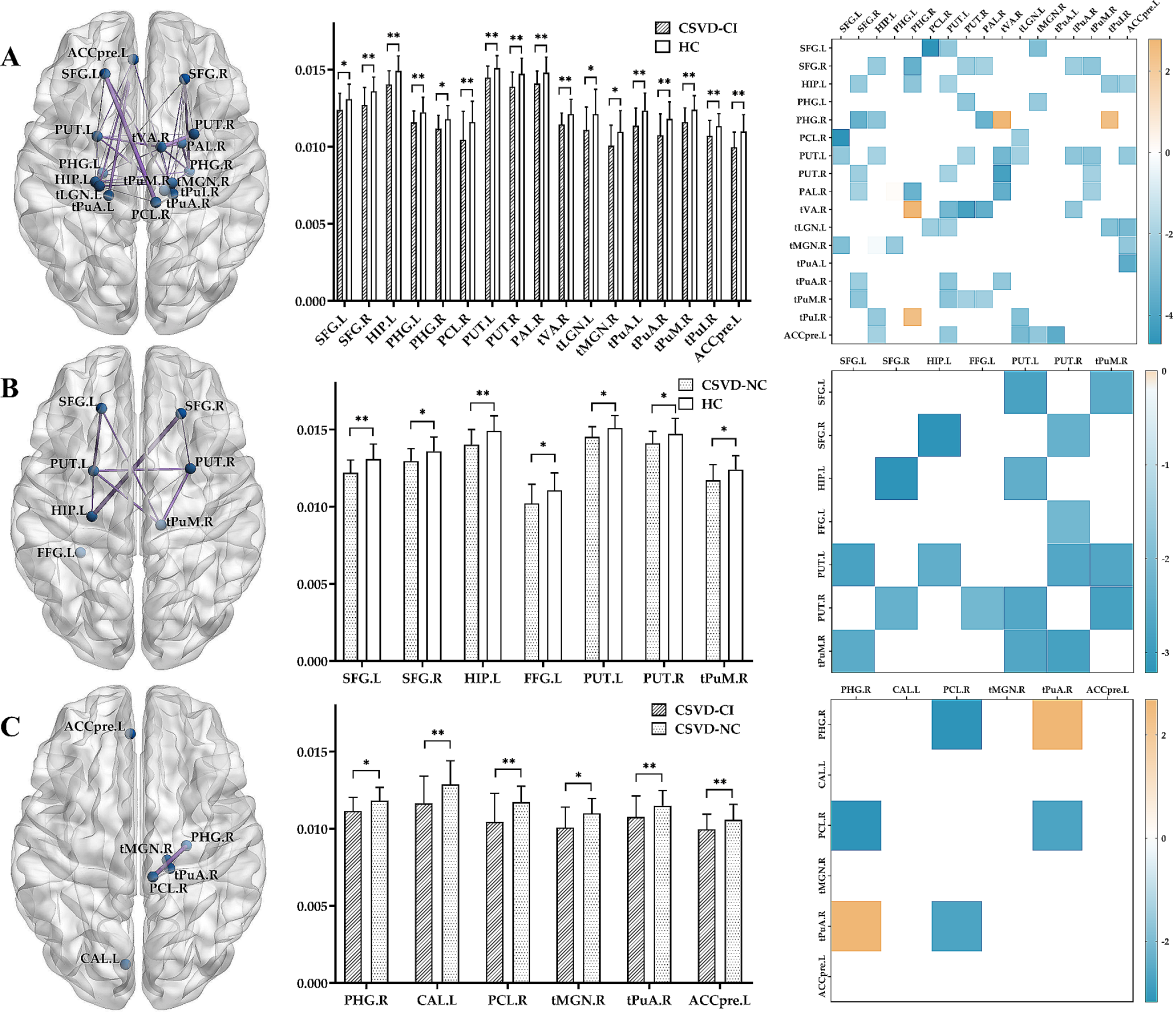
Structural connectome with significant differences between two groups, (**A**) CSVD-CI with HC, (**B**) CSVD-NC with HC, and (**C**) CSVD-CI with CSVD-NC. The first row demonstrates altered regions and connections of structural networks. Nodes represent brain areas with remarkably different values of nodal efficiency, and edges represent connections between differential brain regions. The second row demonstrates significantly differential regions of nodal efficiency. *, *p* < 0.05 for comparison between groups; **, *p* < 0.01. In the third row, the heat maps demonstrate structural connections between the two brain regions and their strength. ACCpre, pregenual of anterior cingulate cortex; CAL, calcarine fissure and surrounding cortex; FFG, fusiform gyrus; HIP, hippocampus; PAL, pallidum; PCL, paracentral lobule; PHG, parahippocampal gyrus; PUT, putamen; SFG, superior frontal gyrus; tLGN, lateral geniculate thalamus; tMGN, medial geniculate thalamus; tPuA, pulvinar anterior thalamus; tPuI, pulvinar inferior thalamus; tPuM, pulvinar medial thalamus; tVA, ventral anterior thalamus; L, left; R, right; CSVD-CI, CSVD patients with cognitive impairment; CSVD-NC, CSVD patients with normal cognition; HC, healthy control

Nineteen brain regions had significantly different nodal efficiency among the three groups (*p* < 0.05). Compared with the HC group, the CSVD-NC group demonstrated decreased nodal efficiency in the bilateral dorsolateral superior frontal gyrus (SFG) (L, *p* = 0.004; R, *p* = 0.037), left hippocampus (*p* = 0.003), left fusiform gyrus (*p* = 0.014), bilateral putamen (L, *p* = 0.013; R, *p* = 0.035) and right pulvinar medial thalamus (tPuM) (*p* = 0.020). Besides, the CSVD-CI group further showed considerably decreased nodal efficiency in the bilateral parahippocampal gyrus (PHG) (L, *p* = 0.007; R, *p* = 0.014), right paracentral lobule (*p* = 0.009), right pallidum (*p* = 0.008), right ventral anterior thalamus (tVA) (*p* = 0.008), left lateral geniculate thalamus (tLGN) (*p* = 0.013), right medial geniculate thalamus (tMGN) (*p* = 0.014), bilateral pulvinar anterior thalamus (tPuA) (L, *p* = 0.002; R, *p* = 0.003), right pulvinar inferior thalamus (tPuI) (*p* = 0.008) and left pregenual anterior cingulate cortex (ACCpre) (*p* = 0.001). Moreover, compared with the CSVD-NC group, the CSVD-CI group exhibited significantly decreased nodal efficiency in the right PHG (*p* = 0.010), left calcarine (*p* = 0.007), right paracentral lobule (*p* = 0.004), right tMGN (*p* = 0.012), right tPuA (*p* = 0.035), and left ACCpre (*p* = 0.043) (Table 2; Fig. 1).

**Table 2:** Brain regions showing significantly altered nodal efficiency among three groups

Region	CSVD-CI	CSVD-NC	HC	f	*p*	FDR-*p*	Post hoc analysis		
							*p*	*p*	*p*
							CSVD-CI vs. HC	CSVD-NC vs. HC	CSVD-CI vs. CSVD-NC
SFG.L	1.24 × 10^− 2^±1.06 × 10^− 3^	1.22 × 10^− 2^±8.17 × 10^− 4^	1.31 × 10^− 2^±9.68 × 10^− 4^	5.039	0.009	0.019	0.014	0.004	-
SFG.R	1.27 × 10^− 2^±1.14 × 10^− 3^	1.30 × 10^− 2^±7.94 × 10^− 4^	1.36 × 10^− 2^±9.15 × 10^− 4^	5.341	0.007	0.019	0.002	0.037	-
HIP.L	1.40 × 10^− 2^±8.89 × 10^− 4^	1.40 × 10^− 2^±9.79 × 10^− 4^	1.49 × 10^− 2^±9.77 × 10^− 4^	6.704	0.002	0.019	0.002	0.003	-
PHG.L	1.16 × 10^− 2^±7.49 × 10^− 4^	1.19 × 10^− 2^±8.83 × 10^− 4^	1.22 × 10^− 2^±9.56 × 10^− 4^	3.910	0.025	0.032	0.007	-	-
PHG.R	1.12 × 10^− 2^±8.80 × 10^− 4^	1.18 × 10^− 2^±8.64 × 10^− 4^	1.18 × 10^− 2^±8.84 × 10^− 4^	4.675	0.012	0.021	0.014	-	0.010
CAL.L	1.16 × 10^− 2^±1.77 × 10^− 3^	1.29 × 10^− 2^±1.53 × 10^− 3^	1.23 × 10^− 2^±1.20 × 10^− 3^	4.020	0.022	0.032	-	-	0.007
FFG.L	1.05 × 10^− 2^±9.64 × 10^− 4^	1.02 × 10^− 2^±1.23 × 10^− 3^	1.10 × 10^− 2^±1.12 × 10^− 3^	3.363	0.040	0.040	-	0.014	-
PCL.R	1.04 × 10^− 2^±1.85 × 10^− 3^	1.17 × 10^− 2^±1.04 × 10^− 3^	1.16 × 10^− 2^±1.36 × 10^− 3^	5.665	0.005	0.019	0.009	-	0.004
PUT.L	1.45 × 10^− 2^±7.31 × 10^− 4^	1.45 × 10^− 2^±6.52 × 10^− 4^	1.51 × 10^− 2^±8.08 × 10^− 4^	4.906	0.010	0.019	0.005	0.013	-
PUT.R	1.39 × 10^− 2^±9.38 × 10^− 4^	1.41 × 10^− 2^±7.75 × 10^− 4^	1.47 × 10^− 2^±1.02 × 10^− 3^	5.059	0.009	0.019	0.003	0.035	-
PAL.R	1.41 × 10^− 2^±8.07 × 10^− 4^	1.44 × 10^− 2^±9.43 × 10^− 4^	1.48 × 10^− 2^±1.01 × 10^− 3^	3.744	0.029	0.032	0.008	-	-
tVA.R	1.14 × 10^− 2^±7.54 × 10^− 4^	1.18 × 10^− 2^±9.78 × 10^− 4^	1.21 × 10^− 2^±9.54 × 10^− 4^	3.841	0.026	0.032	0.008	-	-
tLGN.L	1.11 × 10^− 2^±1.49 × 10^− 3^	1.18 × 10^− 2^±1.17 × 10^− 3^	1.21 × 10^− 2^±1.60 × 10^− 3^	3.431	0.038	0.040	0.013	-	-
tMGN.R	1.01 × 10^− 2^±1.32 × 10^− 3^	1.10 × 10^− 2^±9.61 × 10^− 4^	1.10 × 10^− 2^±1.38 × 10^− 3^	4.599	0.013	0.021	0.014	-	0.012
tPuA.L	1.14 × 10^− 2^±1.14 × 10^− 3^	1.20 × 10^− 2^±9.44 × 10^− 4^	1.24 × 10^− 2^±1.13 × 10^− 3^	5.396	0.007	0.019	0.002	-	-
tPuA.R	1.08 × 10^− 2^±1.37 × 10^− 3^	1.15 × 10^− 2^±9.85 × 10^− 4^	1.18 × 10^− 2^±1.11 × 10^− 3^	5.249	0.008	0.019	0.003	-	0.035
tPuM.R	1.16 × 10^− 2^±9.28 × 10^− 4^	1.17 × 10^− 2^±1.00 × 10^− 3^	1.24 × 10^− 2^±9.10 × 10^− 4^	5.077	0.009	0.019	0.003	0.020	-
tPuI.R	1.07 × 10^− 2^±9.96 × 10^− 4^	1.08 × 10^− 2^±1.06 × 10^− 3^	1.13 × 10^− 2^±8.14 × 10^− 4^	3.815	0.027	0.032	0.008	-	-
ACCpre.L	9.97 × 10^− 3^±9.83 × 10^− 4^	1.06 × 10^− 2^±1.01 × 10^− 3^	1.10 × 10^− 2^±1.10 × 10^− 3^	6.328	0.003	0.019	0.001	-	0.043

***Note***: *p* < 0.05, LSD-corrected

ACCpre, pregenual of anterior cingulate cortex; CAL, calcarine; FFG, fusiform gyrus; HIP, hippocampus; PAL, pallidum; PCL, paracentral lobule; PHG, parahippocampal gyrus; PUT, putamen; SFG, superior frontal gyrus; tLGN, lateral geniculate of thalamus; tMGN, medial geniculate of thalamus; tPuA, pulvinar anterior of thalamus; tPuI, pulvinar inferior of thalamus; tPuM, pulvinar medial of thalamus; tVA, ventral anterior of thalamus; L, left; R, right; CSVD-CI, CSVD patients with cognitive impairment; CSVD-NC, CSVD patients with normal cognition; HC, healthy control

The connectivity alterations could be observed in the heat map (Fig. 1). Most of the structural connections were decreased in both patient groups compared with the HC group. Nevertheless, the network connections significantly increased between right PHG and right tVA and tPuI in the CSVD-CI group compared with the HC group and between the right PHG and right tPuA in the CSVD-CI group compared with the CSVD-NC group.

**Fig. 2:**
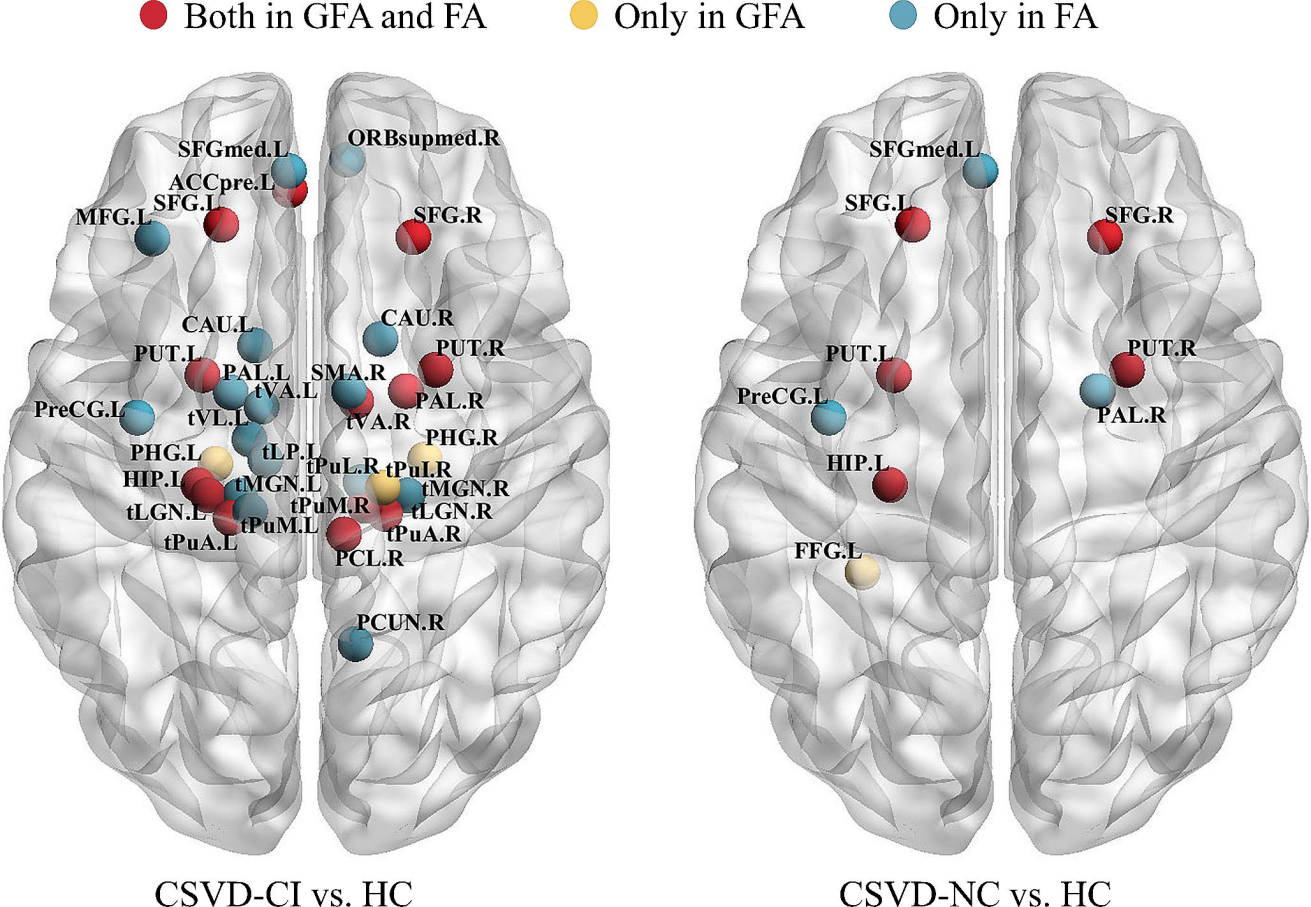
Structural connectome with significant differences between two groups respectively based on GFA and FA. ACCpre, pregenual of anterior cingulate cortex; CAU, caudate nucleus; FFG, fusiform gyrus; HIP, hippocampus; MFG, middle frontal gyrus; ORBsupmed, superior frontal gyrus, medial orbital; PAL, pallidum; PCL, paracentral lobule; PCUN, precuneus; PHG, parahippocampal gyrus; PreCG, precentral gyrus; PUT, putamen; SFG, superior frontal gyrus; SFGmed, superior frontal gyrus, medial; SMA, supplementary motor area; tLGN, lateral geniculate thalamus; tLP, lateral posterior thalamus; tMGN, medial geniculate thalamus; tPuA, pulvinar anterior thalamus; tPuI, pulvinar inferior thalamus; tPuL, pulvinar lateral thalamus; tPuM, pulvinar medial thalamus; tVA, ventral anterior thalamus; tVL, ventral lateral thalamus; L, left; R, right; CSVD-CI, CSVD patients with cognitive impairment; CSVD-NC, CSVD patients with normal cognition; HC, healthy control

We performed brain network analysis based on FA using the same pipeline and compared with GFA. There was a good consistency between the results of FA and GFA, with 16 overlapping brain regions (Fig. 2). However, there were some regions existing only in FA, including the left precentral gyrus, left middle frontal gyrus, right supplementary motor area, left medial superior frontal gyrus, right superior frontal gyrus, medial orbital, right precuneus, bilateral caudate nucleus, left pallidum, left lateral posterior thalamus, left tVA, left ventral lateral thalamus, right tLGN, left tMGN, left tPuM, and right pulvinar lateral thalamus. The additional regions using GFA included the bilateral PHG, left fusiform gyrus, and right tPuI.

### Global network analysis

Increased aLp (*p* = 0.024) and decreased aEglob (*p* = 0.020) based on GFA were observed in the CSVD-CI and CSVD-NC group compared with the HC group (Table 3). All three groups of brain structural networks conformed to small-world properties with γ > 1.1, λ ≈ 1, and σ > 1.

**Table 3:** Global network analysis among three groups

	CSVD-CI	CSVD-NC	HC	*p*
aEglob	1.041 × 10^− 2^±4.098 × 10^− 4^	1.052 × 10^− 2^±4.276 × 10^− 4^	1.076 × 10^− 2^±4.760 × 10^− 4^	**0.020** ^**#**^
aLp	3.887 ± 0.161	3.841 ± 0.155	3.758 ± 0.172	**0.024** ^**#**^

Note: Data represented as mean ± SD

^**#**^ indicates significant differences between CSVD-CI group and HC group.

aEglob, AUC value of global efficiency; aLp, AUC value of characteristic path length; CSVD-CI, CSVD patients with cognitive impairment; CSVD-NC, CSVD patients with normal cognition; HC, healthy control

### Correlation analysis

**Fig. 3:**
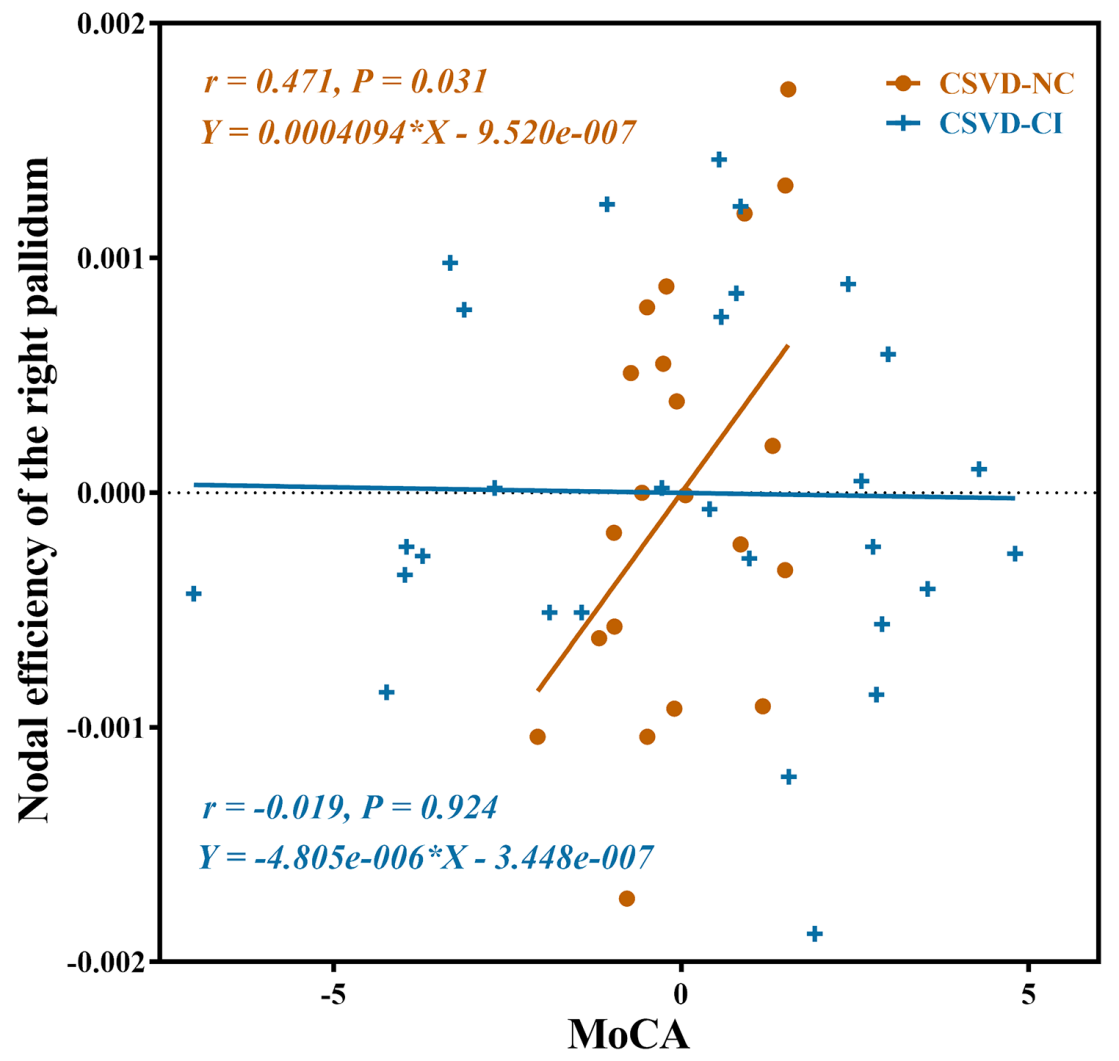
The correlation between nodal efficiency of the right pallidum and MoCA in CSVD-NC and CSVD-CI groups. Of note, the coordinates of both the X axis (MoCA) and Y axis (nodal efficiency of the right pallidum) do not reflect the initial values of these variables when taking age, sex and education as covariates. MoCA, the Montreal Cognitive Assessment

The nodal efficiency in the right pallidum was positively associated with MoCA in the CSVD-NC group (*r* = 0.471, *p* = 0.031) (Fig. 3). No significant correlations were observed between global network parameters and MoCA.

## Discussion

The present study explored the subtle changes in structural connectivity in patients with CSVD. First, the brain regions with decreased nodal efficiency and disrupted structural connectivity were predominantly concentrated in corticothalamic pathways in both patient groups. Second, structural connectivity increased between the right PHG and thalamus subregions in the CSVD-CI group, compared with the CSVD-NC and HC groups. Finally, the global topological organization in CSVD patients was disrupted, as indicated by decreased global efficiency and increased characteristic path length.

Damage to regions in CSVD patients may be associated with cognitive decline and can make these regions more susceptible to disease. Based on GFA and FA, brain regions exhibiting significantly decreased nodal efficiency were primarily concentrated in the thalamus, frontal lobes, hippocampus, and basal ganglia in both patient groups compared with HCs. Notably, the efficiency of information processing in brain regions within the corticothalamic pathways reduced in vascular cognitive decline (Jellinger, 2007). These pathways consisted of interconnected networks responsible for processing decision-making, cognition, associative, and sensorimotor information (Fischer, 2021; Vich et al., 2022). In addition, amyloid deposition is believed to contribute to the impairment of information transmission and to have a synergistic effect with structural brain disruption. Together, these factors can lead to extensive breakdown of brain tissue, eventually leading to cognitive impairment (Son et al., 2022). There were more regions decreased in FA, which might indicate the notion that FA values were underestimated in the regions containing crossing fibers (Winston, 2012). Moreover, the locations of these brain regions were also consistent with previous studies, focusing on the centrum semiovale, superior longitudinal fasciculus, and thalamus (Oouchi et al., 2007; Szeszko et al., 2018). The results based on GFA could be more sensitive than FA due to observation of the PHG and FFG, which suggested us to focus on the role of these brain regions in CSVD patients.

Although a few CSVD patients maintain normal cognition, they might still experience structural and functional alterations. We speculated that decreased nodal efficiency in corticothalamic pathways could serve as an early indicator of CSVD-related cognitive decline. The nodal efficiency of the right pallidum was positively associated with MoCA in CSVD-NC patients. The pallidum plays a pivotal role as a “transfer station” connecting the cortex to the thalamus to participate in cortical regulation, including motor control, associative learning, and planning (Obeso et al., 2008). Our findings supported the role of the pallidum in cognition of CSVD patients. Furthermore, the CSVD-CI group exhibited more regions with substantial decreases in nodal efficiency compared with the CSVD-NC and HC groups. These regions, including the left PHG, right pallidum, right tVA, left tLGN, left tPuA, and right tPuI, were part of the subcortical network. This network is pivotal in facilitating large-scale neural communication, with the basal ganglia and thalamus playing crucial roles as part of a core circuit supporting the integration of neural information (Bell & Shine, 2016). In the current study, the disrupted corticothalamic structural connectivity in patients with CSVD might suggest the potential pathophysiological mechanisms in this disease.

This study demonstrated that corticothalamic dysconnectivity was associated with global cognition (Chen et al., 2019). Of note, there were increased connections between the right PHG and right striato-thalamic regions in the CSVD-CI group, which may be a compensation or reorganization between networks (Filippi & Rocca, 2003). The PHG, a part of memory system, is involved in complex cognitive performance (Kesslak et al., 1991). The striato-thalamic pathway is critical for information transmission from cortex and subcortex. Ter Telgte et al. suggested that the efficiency of structural networks was a form of brain reserve preventing clinical deterioration. The present study demonstrated that the brain could use alternative connectivity or connection enhancement to compensate for the disruption of white matter microstructure.

This study observed that both CSVD patients and the HCs conformed to the small-worldness, which was consistent with the previous studies based on FA or fiber number (Wen et al., 2017; Xin et al., 2022). The small-world network aims to maximize efficiency and minimize cost (Bassett & Bullmore, 2006). However, small-worldness does not fully reflect and generalize the complex brain network (Bassett & Bullmore, 2017). Consistent with a longitudinal demonstrating study that network efficiency at baseline predicted mortality and cognitive decline (Tuladhar et al., 2020), a decrease in global efficiency and an increase in characteristic path length indicated connectivity alterations in both patient groups compared with HCs. This might imply a decline in information transfer efficiency and functional integration ability among brain regions in this disease.

This study had several limitations. First, MoCA reflects only overall cognition. Comprehensive neuropsychological tests will be required in the future to explore deficits specific to sub-domains. Second, the sample size was relatively small. Future studies with larger sample sizes are needed to validate the current findings. Third, the analyses were cross-sectional, which made it difficult to detect temporal and causal relationships among the variables. Hence, longitudinal studies are necessary to explore the pattern of disease evolution.

## Conclusions

This study demonstrated that efficient communication in corticothalamic pathways was disturbed in CSVD patients, of which the structural connectivity was strongly affected in CSVD-CIs. The extensive disruption of structural network provided evidence for the consideration of CSVD as a global brain disease. We also found the compensation phenomenon in CSVD-CIs and provided evidence for a role of the right pallidum in cognition in CSVD-NCs.

## Data Availability

Not applicable.

## Declarations

This study was approved by the Beijing Chaoyang Hospital Ethics Review Board. It met the guidelines of Capital Medical University, which abides by the Helsinki Declaration on ethical principles for medical research involving human participants. Informed consent was obtained from all participants involved in the study.
